# Combining Chromatographic, Rheological, and Mechanical Analysis to Study the Manufacturing Potential of Acrylic Blends into Polyacrylic Casts

**DOI:** 10.3390/ma14226939

**Published:** 2021-11-17

**Authors:** Pablo Reyes, Mariya Edeleva, Dagmar R. D’hooge, Ludwig Cardon, Pieter Cornillie

**Affiliations:** 1 Laboratory of Morphology, Faculty of Veterinary Sciences, Ghent University, Salisburylaan 133, 9820 Merelbeke, Belgium; pablo.reyesisaacura@ugent.be; 2 Centre for Polymer and Material Technologies (CPMT), Department of Materials, Textiles and Chemical Engineering, Ghent University, Technologiepark 130, Zwijnaarde, 9052 Ghent, Belgium; ludwig.cardon@ugent.be; 3 Laboratory for Chemical Technology (LCT), Department of Materials, Textiles and Chemical Engineering, Ghent University, Technologiepark 125, Zwijnaarde, 9052 Ghent, Belgium; mariya.edeleva@ugent.be; 4 Centre for Textiles Science and Engineering (CTSE), Department of Materials, Textiles and Chemical Engineering, Ghent University, Technologiepark 70A, Zwijnaarde, 9052 Ghent, Belgium

**Keywords:** polymeric material characterization, coformulations design, corrosion casting

## Abstract

Polyacrylics have been considered for a broad range of material applications, including coatings, dental applications, and adhesives. In this experimental study, the casting potential of a group of (co)monomers belonging to the acrylic family has been explored to enable a more sustainable use of these polymer materials in the medical and veterinary science field. The individual contributions of each comonomer have been analyzed, the reaction conversion has been studied via gas chromatography (GC), the rheological behavior has been characterized via stress-controlled measurements, and the final mechanical properties have been obtained from tensile, flexure, and impact tests. The GC results allow assessing the pot life and thus the working window of the casting process. For the rheological measurements, which start from low-viscous mixtures, a novel protocol has been introduced to obtain accurate absolute data. The rheological data reflect the time dependencies of the GC data but facilitate a more direct link with the macroscopic material data. Specifically, the steep increase in the viscosity with increasing reaction time for the methyl methacrylate (MMA)/ethylene glycol dimethyl methacrylate (EGDMA) case (2% crosslinker) allows maximizing several mechanical properties: the tensile/flexure modulus, the tensile/flexure stress at break, and the impact strength. This opens the pathway to more dedicated chemistry design for corrosion casting and polyacrylic material design in general.

## 1. Introduction

Polymers are well known for their wide range of applications, including both daily products and specific polymers for niche markets. Amongst these polymers, poly (methyl methacrylate), or shortly PMMA, has a significant role in the polymer industry [1,2,3,4,5]. This has become further evident during the current COVID pandemic through the utilization of PMMA for personal protective equipment [6,7,8,9,10,11]. PMMA is a good glass substitute thanks to its mechanical properties and transparency [1,3,12,13]. It also possesses chemical recycling potential, contributing to its circularity [14,15,16,17,18,19,20]. In the medical field, PMMA is the main constituent of bone cement [21,22,23,24,25]. PMMA is also the main component of resins used to make dental prosthetics and dentures [1,2,3,26,27,28,29,30]. 

Another PMMA-based application is so-called corrosion casting, which anatomists use to create models of the space inside body systems or elements [31,32,33,34,35,36,37]. Examples are the vascular, the respiratory, and the lymphatic system that can be filled with a resin that solidifies, therefore creating a replica [31,34,38,39,40,41,42,43,44,45]. Polymeric resins are commonly used for this purpose, as they have been successful in offering a reasonable solidification time [31,32,46,47] and an acceptable resistance to corroding agents [32,48]. Nonetheless, several characteristics still need to be further improved: (1) the pot-time, or the lifetime that the resin mixture can be applied [31,34,40,44,46,49], (2) the rheology/flow, as this defines the performance in filling [31,39,48,49,50,51,52,53], and (3) the durability of the resistance, as the final cast has to be resistant for subsequent manipulation and study [34,39,51,53,54,55]. In any case, there is a strive for more sustainable PMMA manufacturing processes. This can be either on the level of the blend/polymer material design or through the more efficient exploitation of the (polymer) processing technique delivering the final material product.

**Figure 1:**
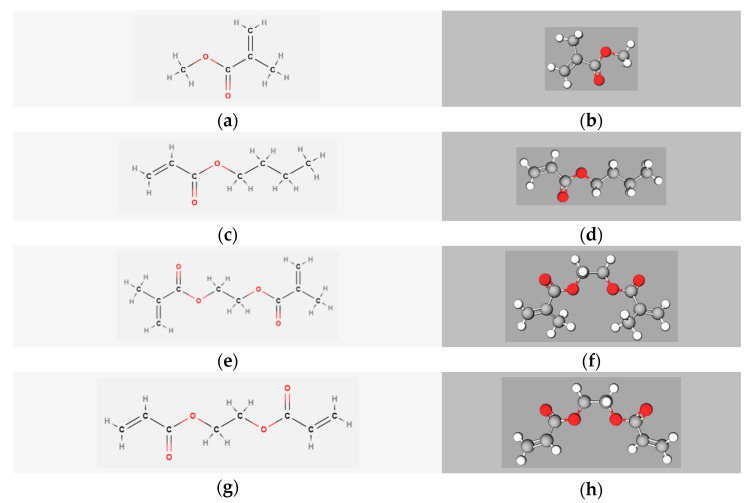
Chemical representations of Methyl Methacrylate (**a**,**b**), *n*-Butyl Acrylate (**c**,**d**), Ethylene Glycol Dimethacrylate (**e**,**f**), and Ethylene Glycol Diacrylate (**g**,**h**). The left side corresponds to a structural model of the chemical formula, while the right side corresponds to a 3D model based on spheres and sticks.

For the (corrosion) casting process, the main process variable is the chemical formulation, and in general, acrylic-based resins are utilized that are more complex than simply Methyl Methacrylate (MMA). MMA often occupies the role of the key monomer, but in practice, one adds comonomers from the same monomer family, hence, methacrylates, or from a compatible monomer family being acrylates [56,57,58,59,60]. Figure 1 displays the chemical representations of three monomers (alongside MMA) relevant in this coformulation design: *n*-Butyl Acrylate (BA), Ethylene Glycol Dimethacrylate (EGDMA), and Ethylene Glycol Diacrylate (EGDA). The chemical formula is displayed as a structural model on the left side, while a 3D model based on spheres and sticks is shown on the right side. All four molecules in Figure 1 possess a vinyl group directly attached to the carbon of an ester group. BA is widely used as a comonomer to enhance flexibility and increase impact strength [1,22,28]. EGDMA and EGDA have two vinyl groups, which allows them to form crosslinked systems and ultimately networks and to also strengthen the polymer mechanical properties [25,30,61,62,63]. 

A limited number of studies have systematically investigated the relationship between the acrylic blend proportions and final material/macroscopic properties. For instance, corrosion casting studies with MMA-based resins have been limited to the investigation of the effect of diluting the resin [35,46,64]. To the best knowledge of the authors, the first casting MMA-based resins were inspired by the resins developed for dental applications so that casting resins consisted of liquid MMA, PMMA powder, and some initiator [40], alongside additives. Thus, variations including the building blocks from Figure 1 are lacking and a key novelty, as explored in the present work, is the consideration of such variations. 

Previous research on dental materials and other closely related MMA-based materials such as bone cement provides at most only complementary information in connection to research on (corrosion) casting materials. There is specifically a strong desire for more dedicated rheological measurements [65,66,67,68,69,70,71]. Mechanical properties have been also measured for MMA-based materials [72,73,74,75,76], but emphasis is typically on one mechanical property. 

Therefore, the current experimental study seeks to screen more systematically the chemistry, rheology, and mechanical properties of acrylic coformulations (Figure 1) in view of their (corrosion) casting potential. Individual comonomer contributions are assessed to correlate the relevance of the coformulation with both the behavior of the system during polymerization and the quality of the final product, the latter through the production of test specimens through well-designed silicone molds. To facilitate the bridging of chemistry with final applications, a novel rheological protocol is introduced that is capable of reliably treating data recording, starting from an improved blending strategy and a low viscous mixture. 

It is demonstrated that the pot life is a key manufacturing parameter, and a maximization of tensile, flexure, and impact macroscopic properties can be associated with a strong increase in viscosity data, highlighting the relevance of the novel rheological protocol.

## 2. Materials and Methods

### 2.1. (Co)monomers

Methyl Methacrylate (MMA), *n*-Butyl Acrylate (BA), and Ethylene Glycol Dimethacrylate (EGDMA) were purchased from Sigma-Aldrich brand and provided by CHEMLAB ANALYTICAL bvba, Zedelgem, Belgium. Ethylene Glycol Diacrylate (EGDA) was purchased from Acros-Organics brand and provided by VWR International, LLC., Leuven, Belgium. Every monomer bottle contained methyl ethyl hydroquinone (MEHQ) as a polymerization inhibitor. Each monomer was passed through a bed of aluminum oxide to remove this inhibitor [77]. The obtained solutions were bottled and stored at 4 °C.

### 2.2. Initiator

Benzoyl Peroxide (BPO), in the commercial form of Luperox^®^ A75, was purchased from Sigma Aldrich brand, provided by Merck Life Science BV, Overijse, Belgium and recrystallized from a solution in acetone [78].

### 2.3. Accelerator

N,N-bis-(2-Hydroxyethyl)-p-Toluidine (DHEPT) was purchased from Aldrich Chemistry brand, provided by Merck Life Science BV, Overijse, Belgium, and recrystallized from a solution in cyclohexane-toluene (1:1) [79].

### 2.4. Other Substances

Aluminum Oxide (Al_2_O_3_; activated; neutral, Brockmann I) and Hydroquinone with a purity ≥99.5% were purchased from Sigma-Aldrich brand, provided by Merck Life Science BV, Overijse, Belgium. Dichloromethane HPLC grade and Acetone with a purity ≥99.5% were purchased from Chem-Lab NV brand and provided by CHEMLAB ANALYTICAL bvba, Zedelgem, Belgium. *n*-Decane with a purity ≥99% was purchased from Honeywell brand and provided by Thermo Fisher Scientific Inc., Merelbeke, Belgium. All these substances were used as purchased.

### 2.5. Coformulations/Blends and Flask Polymerization Experiments

For every formulation case shown in Table 1, the same overall protocol was followed. An initial comonomer solution was prepared by mixing the comonomers, with the limiting case of only one monomer also included (entry 1). Then, two solutions were prepared by splitting the comonomer solution (or monomer solution for entry 1) as 80 volume % with accelerator being added afterwards (“accelerator solution”) and 20 volume % with initiator being added afterwards (“initiator solution”).

The accelerator solution was split in aliquots of 0.4 mL in glass vials with an open-top cap and septum. The initiator solution was kept in a flask with a neck and closed with a septum. Every solution was sparged and flushed with high-purity argon gas for at least 30 min. Upon checking that the argon atmosphere was preserved, 0.1 mL of the initiator solution was injected into each vial at room temperature (except for entry 1). Upon injection, a stopwatch was started to measure the reaction time. The polymerization was completed in the different vials at different predefined time intervals to enhance the accuracy of each single data point. This polymerization stopping was achieved by immersing the vials in liquid nitrogen for 60 s and then adding a solution of hydroquinone (inhibitor) in acetone/dichloromethane (1:49 in volume).

Note that also a conventional free radical polymerization (FRP) case has been included in Table 1 (Case 1) in which the polymerization temperature is set well above room temperature. Under such conditions, the role of the accelerator is negligible.

### 2.6. Gas Chromatography (GC) Analysis and Conversion Calculations

Chromatographical tests were carried on a TRACE™ GC ULTRA Gas Chromatograph (GC) from Thermo Scientific equipped with an HP-5MS column (30 m × 0.250 mm) from Agilent Technologies Belgium S.A./N.V., Diegem, Belgium. The GC was connected with a flame ionization detector with hydrogen as carrier gas at a flow rate of 1.5 mL·min^−1^. The signal was acquired and recorded with the equipment software, and then analyzed. The (molar) ratios of MMA to *n*-decane were determined by comparing the corresponding area under the signal peak of each compound [80,81]. The MMA conversion profile was calculated from the ratios at different reaction times compared to the ratio at time zero. Before injection in the GC unit, each sample was consistently diluted with dichloromethane and a standard aliquot of *n*-decane as a reference marker [82]. For Cases 5 and 6 in Table 1, BA peaks were also acquired and a similar procedure was used to calculate the BA conversion. Similarly, for the crosslinkers, conversion values have been recorded.

### 2.7. Polymerization in a Rheometer and Rheological Analysis

Rheological tests were performed with an MCR 702 MultiDrive Rheometer from Anton-Paar Benelux BV, Gentbrugge, Belgium. The accelerator solution and the initiator solution were mixed, and a stopwatch was started to measure the preparation time related to transfer. The mix was transferred to the cup of a concentric cylinder (CC) geometry. Then, the cylinder spindle was lowered to the measuring position, and a constant shear rate test was performed. The stopwatch measurement was ended once the rheometer started its test, and this was logged as the finalization of the preparation time. Rheological tests were performed in triplicate format at shear rates of 1 s^−1^, 3 s^−1^, 10 s^−1^, and 80 s^−1^. Each test was performed with an initial temperature of 20 °C with no temperature control (non-isothermal case) and at a controlled temperature of 20 °C (isothermal case).

**Figure 2:**
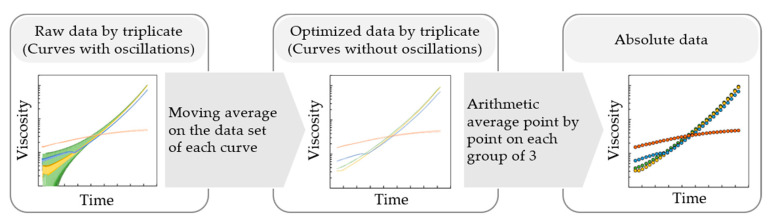
Protocol for the rheological data processing at various shear rates, starting from low viscous mixtures that are reacting and becoming more viscous. The figure shows an example of the raw oscillatory data (**left**), then the moving average step that yields optimized data (**center**), and finally the arithmetic average step that yields the absolute data (**right**) per shear rate value.

Each experiment yielded a table of viscosity data vs. time, as registered with the rheometer software. These data were exported to MS Excel, and the initial (reaction) time was corrected by adding the preparation time measured with the stopwatch. For every raw data set of viscosity values, a moving average was applied over a certain periodic number of points. This process yielded another data set in which the trend is revealed from the oscillatory pattern. In the next step, each group of curves, corresponding to the same value of shear rate, was arithmetically averaged to obtain one (absolute) data set per shear rate value. This dedicated treatment, which to the best of the author’s knowledge has not been reported or developed before, is illustrated in Figure 2. Figures S1–S4 of the Supporting Information highlight the specific individual data treatments in more detail. Hence, the present work puts forward an elegant protocol toward the analysis of rheological data for polymerization reactions with low viscous reagents.

### 2.8. Mechanical Property Tests

**Figure 3:**
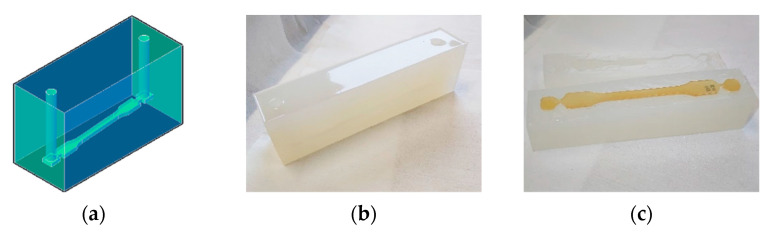
Molds developed for the casting of a polymerizing mixture with low initial viscosity and subsequent shrinkage: (**a**) conceptual representation of the structure of the inside cavity, (**b**) picture of a closed silicone mold, and (**c**) picture of an open silicone mold with a final specimen.

Molds were fabricated for the casting of mechanical testing specimens from a polymerizing mixture with initial low viscosity and volume shrinkage in a later stage. These molds were made in semitransparent silicone and cut open in two parts, which allows opening the molds for the removal of the fully cured specimens. As illustrated in Figure 3, each mold is a solid prism with an inner cavity that is shaped for the targeted specimen. This cavity lies inside the mold with an inclination, minimizing the entrapment of possible air bubbles. Beyond the specimen-shaped cavity ends, the mold system extends longitudinally in both directions: first in narrow necks and then further into short chambers. Over these chambers, two vertical channels connect with the top surface of the mold, as is also clear from Figure 3. 

The molds were cleaned, dried, and fixed in preparation for the casting. The accelerator solution and the initiator solution were mixed, and the resulting mixture was poured through the channel connecting to the lowest chamber until the liquid level reached the top of the opposite channel. Both channels were covered to minimize contact with air. The specimens were removed from the molds after a minimum of 24 h, after which they were allowed to rest for at least 3 days on a flat surface to ensure maximum curing before proceeding further.

Dog bone type 1A specimens (see Figure 3) were produced according to ISO 527-2 standard for tensile testing. In addition, rectangular un-notched type 1 specimens were prepared according to the ISO 197-1 standard for the flexure test and the Charpy impact test. Specimens were stabilized in a controlled temperature and humidity environment for at least 48 h before testing. Tensile tests were conducted according to the ISO 527 standard on an Instron 5565 testing machine, from Instron, using a strain rate of 1 mm min^−1^. Flexure tests were conducted according to the ISO 178 standard on an Instron 4464 testing machine, also from Instron, using a flexion rate of 1 mm min^−1^. Impact tests were conducted according to ISO 179 standard on an IT 503 machine from Tinius Olsen, with an initial pendulum energy of 2.75 J.

## 3. Results and Discussion

In this section, emphasis is first on the analysis of the GC data to then distill interesting coformulations for subsequent rheological analysis and to in a similar manner fine-tune the pool of coformulations for mechanical analysis.

### 3.1. Analysis of GC Data

**Figure 4:**
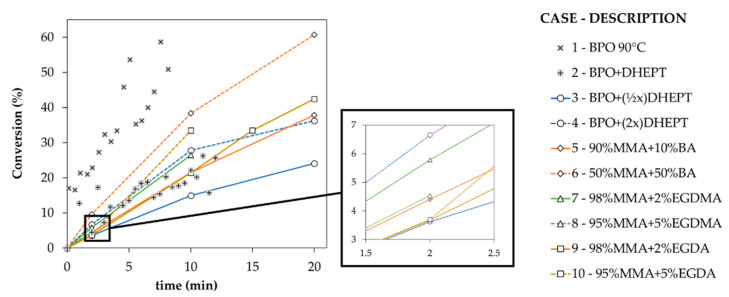
Conversion, as the percentage MMA consumed, versus time for 10 coformulation cases as defined in Table 1. Case 1 starts at 90 °C; other cases at room temperature; full vs. dashed line: lower vs. higher amount.

The results of the conversion vs. time experiments for the cases in Table 1 are shown in Figure 4. Case 1 has been conducted to compare conventional polymerization results, as initiated by thermal decomposition of the initiator at elevated temperature (90 °C), with targeted casting results (other cases in Table 1), as initiated by chemical interactions involving the accelerator at room temperature. The conventional FRP conditions of Case 1 allow a high MMA conversion in a short time, with e.g., already more than 30% monomer conversion after 5 min (gray “x” symbols in Figure 4). Even though the temperature was not registered in Case 1, after a certain amount of reaction time the samples started to form bubbles, which is practically not recommended. A lower but still acceptable polymerization rate is obtained in Case 2 at room temperature with an accelerator, with e.g., 25% MMA conversion after 10 min (gray “+” symbols in Figure 4). Thus, it makes sense to consider the accelerator-triggered initiation of the polymerization at a lower temperature. 

Case 3 (full blue line in Figure 4) and Case 4 (dashed blue line in Figure 4) show the behavior if the proportion of the chemical initiator to accelerator is reduced by half and increased by a factor of 2, compared to Case 2. Such a design is essential to illustrate the impact of the accelerator amount on the MMA consumption rate. The blue lines are more or less embedding the gray “+” symbols of Case 2, highlighting the relevance of this process parameter and the overall reliability of the data set recording. Furthermore, Case 5 and Case 6 (full and dashed orange lines in Figure 4) show the effect of using BA as a comonomer in different proportions to MMA, again with as reference Case 2 having no such BA content. Here, the relative position of the gray “+” symbols is less evident, which is indicative of less trivial chemical interactions. Indeed, the homopolymerization of BA is faster than the homopolymerization of MMA [83], although cross-propagation is favored for the BA-radical/MMA pair compared to the MMA-radical/BA pair, as clear upon comparing the monomer reactivity ratios [84,85,86,87]. In Case 6 (orange dashed line in Figure 4), with a high BA amount, a very high MMA consumption rate is obtained, but for Case 5 with a lower BA amount (orange full line in Figure 4), still, a reasonable consumption rate is observed, although it is not that different compared to Case 2. Thus, it follows that the BA amount is an interesting handle to push the rate forward in a given processing window.

Upon replacing this BA monomer by a crosslinker of either the methacrylate (EGDMA) or acrylate (EDGA) type and using small amounts of this crosslinker (2 or 5%), globally again lower rates are obtained. This is clear upon inspecting the results Cases 7–10 that are associated with the green and ocher lines in Figure 4. These lines globally reflect results closer to the reference Case 2 with no crosslinker and only MMA. An exception is the 5% EDGA crosslinker case (Case 10; dashed ocher lines in Figure 4) with more MMA consumption even close to fast consumption in Case 6 (high BA amount; orange dashed line in Figure 4). This last observation highlights the relevance of the optimization of the type of crosslinker (acrylate over methacrylate) and its amount (5 instead of 2%). Upon using the EDGMA crosslinker with a 5% amount (green dashed lines), the MMA rates are for instance lower, and the crosslinker also slows down the polymerization, again demonstrating the acrylate/methacrylate monomer balance.

**Figure 5:**
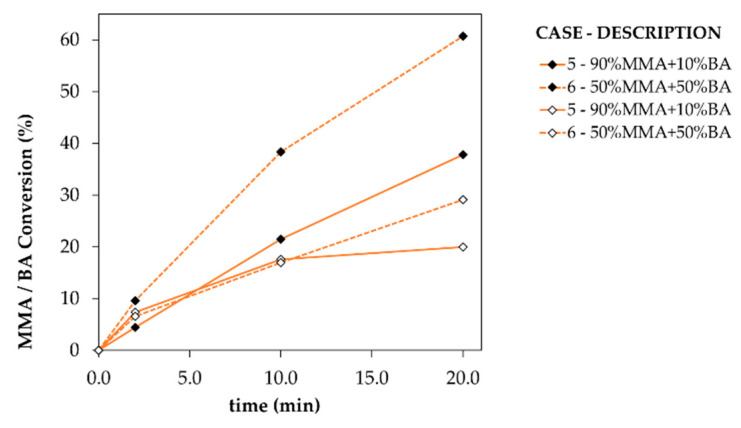
Conversion, as both the percentage MMA consumed (◆) and BA consumed (◇) versus the reaction time for the coformulation cases in Table 1 in which BA is present, namely Cases 5 and 6. Both cases are starting at room temperature.

This strong impact of the use of acrylate monomers is further clear upon inspecting the additional data in Figure 5. In that figure, displaying both the conversion of MMA and BA, a subtle yet important difference between the way MMA and BA are consumed is illustrated. In the visualized timeframe up to 20 min, the MMA conversion increases more rapidly than the BA conversion for both cases (Case 5 and 6 in Table 1), as the closed symbols are located more at the top of Figure 5. Furthermore, for both monomers, the rate of growth decreases as time advances. This is especially true for BA, as evidenced by the more flattening of the lower data points in Figure 5. Multiple (competitive) factors contribute to this effect. One first needs to be reminded that the (intrinsic/chemical) homopropagation reactivity is higher, at least one order of magnitude, for BA. Then, it is necessary to add in the reasoning the impact of diffusional effects. Diffusivity codetermines the reaction rates [88,89], as it can hinder selectively the capacity of the molecules to move and come at a distance to effectively react. Diffusional limitations are significantly more relevant in the (homo)polymerization of MMA, as being the cause of a strong gel effect [26,90,91,92,93], limiting the impact of the termination reaction and yielding an increase in the polymerization rate. Furthermore, reactivity ratios also determine which type of propagation reaction will be favored once the molecules are close enough to react. As already explained above, the reactivity ratios show that whenever there is an active growing chain, being the radical located either in a terminal unit of MMA or BA, the reaction will be favored toward the addition of another MMA unit. These three aspects, coupled with a fourth aspect being the initial limited (or maximum equivalent) availability of BA, explains why on a net basis the rate of growth for the conversion of BA decreases upon comparison with the rate for MMA.

Another interesting research angle is the investigation of the material strength already during the synthesis, which is expected to be higher with more intense crosslinking. For example, Case 3 through Case 6 (all no crosslinker) in Table 1 did not give any issues regarding sampling and could be easily sampled after 2, 10, and 20 min. In contrast, the last sample as recorded at 20 min for Case 7 (with EGDMA crosslinker) cracked once the sample was quenched in liquid nitrogen (first entry in Table 2). After observing this cracking, the following experiments (Cases 8–10; all crosslinker) had an additional sampling point at 15 min. Cases 8 and 10, which correspond to the highest proportions of EGDMA and EGDA crosslinker, also developed temperatures that were high enough to cause cracking of the vials containing the reaction mixture upon quenching in liquid nitrogen. The occurrence of this cracking is shown by the crosses in the second and fourth row of Table 2. To further understand this undesired phenomenon, the solid frozen content could be recovered in one piece and put in dichloromethane in preparation for GC analysis. Nonetheless, the samples with high crosslinking amounts could not be dissolved after 72 h, highlighting the sufficiently crosslinked nature of the polymer formed [94]. Hence, toward the development of casting applications, care should be taken not to increase the crosslinking amount too much. For example, in Case 9 with only 2% of EDGA, no cracking was observed, as also indicated by the third entry in Table 2.

Overall, upon considering the results in Figure 4 and Figure 5, and Table 2, it can be concluded that the formulation variables in Table 1 allow varying the time scales to obtain a certain MMA conversion, opening the pathway of casting design at the chemical level. On top of that, these variations allow obtaining likely different material properties.

### 3.2. Rheological Analysis

Upon inspecting the literature, it is obvious that limited effort has been devoted to the dedicated handling of rheological analysis of corrosion casting. Either no viscosity measurements have been performed or only basic data recording has been conducted [36,39,40,48,51,52]. Hitherto, numerous studies highlight the importance of a viscosity low enough to effectively perfuse the injected resin [31,32,34,36,39,43,48,49,54,55,95,96,97], but no well-defined guidelines are available. In the present contribution, specific focus is therefore on the reporting of the methods utilized to obtain reliable rheological data.

Three practical limitations arise from the rheological tests in view of corrosion casting, as explained in what follows. A first practical limitation is that the stabilization of the rheometer readings can be rated as difficult both at low shear rates and at low viscosity values. This is most clear from the oscillations with a high amplitude on the viscosity readings under such operating conditions (cf. Figure 2, left). Such oscillations can be filtered out to an acceptable extent by the experimental protocol in Figure 2 (left to right) and further illustrated in the Supplementary Information. Oscillations are fortunately also reduced as the viscosity builds up during polymerization, and they are smaller with higher values of shear rate. The oscillations are inherent to the equipment working principles. A control system based on shear stress control is characteristic of the rheometer used in the present work. For a test at a constant shear rate, the shear stress has to be adjusted by the control system. The initial low viscosity of the fluid yields a low response that makes it difficult for the equipment to control the shear stress. The equipment could eventually stabilize if the fluid was not also undergoing a polymerization, which makes the viscosity change over time and thus makes the stabilization point a variable. This explains our desire to use a concentric cylinder (CC) geometry. This geometry possesses a large surface that enhances the response, minimizing most of this first practical limitation.

A second limitation is the volume reduction, which is an intrinsic feature of polymerization due to a difference in the density of the monomer and polymer [98], leading to a difficult constraint for geometries requiring a constant sample size. In addition, here, the CC geometry is relevant. If it is operated with a sufficiently high liquid level, it has acceptable tolerance to shrinkage, solving the second limitation.

A third limitation is that experiments have been conducted only to a maximum value of viscosity of 1000 mPa·s because above this point, the geometry cleaning becomes difficult. The time to reach that upper viscosity value is shown in Table 3, selecting four cases from Table 1 that address the most relevant variations in Figure 4, namely a reference case with only MMA (Case 3), one case with MMA and BA (Case 5), and two cases of MMA with respectively the methacrylate and acrylate-based crosslinker (Cases 7 and 9). A further distinction is made between rheological measurements with and without temperature control. Without temperature control, the exothermicity effect increases the polymerization rate and, in that way, the viscosity increases are faster compared to the situation with temperature control. Without temperature control, solidification is reached more easily, although the relative time changes in Table 3 are still moderate (e.g., 10%). This solidification of the polymerizing mixture is a practical difficulty, but it also allows estimating the pot life of the mixture. This pot life is defined as the time that a polymerizing mixture can be manipulated for its application. Therefore, a quick correlation between the times shown in Table 3 and the pot life is possible. As it is difficult to clean mixtures with too high viscosity, accordingly, a casting mixture would be also difficult to manipulate or to be injected into a cavity for a similar timing.

**Figure 6:**
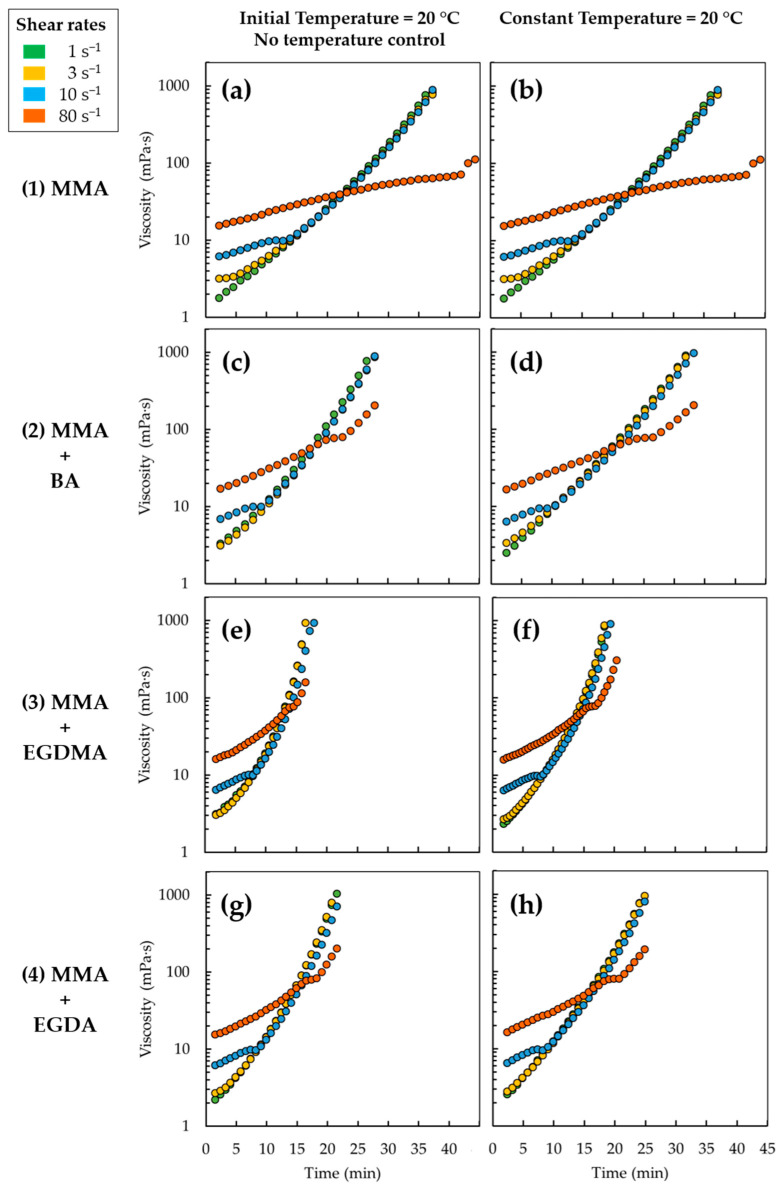
Curves of viscosity versus reaction time at four shear rates (1 s^−1^ in green, 3 s^−1^ in yellow, 10 s ^−1^ in blue, and 80 s^−1^ in orange), for four coformulations (from top to bottom) (**1**) MMA (subplots (**a,b**)), (**2**) MMA + BA (9:1) (subplots (**c**,**d**)), (**3**) MMA + EGDMA (98:2) (subplots (**e**,**f**)), (**4**) MMA + EGDA (98:2) (subplots (**g**,**h**)) (Cases 3, 5, 7, and 9 in Table 1). Every test case was evaluated under two types of conditions: initial temperature of 20 °C followed by no temperature control (left column), and constant temperature of 20 °C (right column). All data are reported after the application of protocol in Figure 2.

As shown in Figure 6, for every case in Table 3, the viscosity increases with time, as expected for a polymerization. Each curve in this figure exhibits an exponential shape over a large part of the time variation, at least with a vertical logarithmic scale. This logarithmic vertical scale visually smoothens the steepness of the growth but also allows distinguishing the details that otherwise would be invisible at the early stages of polymerization. Consistent with the results in Table 3, the viscosity results under isothermal conditions, as displayed in the right column in Figure 6, are relatively lower compared to the left column results in Figure 6 (no temperature control) due to less pronounced gel effects.

More in detail, it follows from Figure 6 that the green curves corresponding to a shear rate of 1 s^−1^ grow exponentially over the whole time range. For a shear rate of 3 s^−1^, there is a small zone at the beginning in which the yellow curve is convex upward, and then, the curve form becomes exponential and overlaps the curve of 1 s^−1^. For a shear rate of 10 s^−1^, there is also a convex zone (blue curves) that extends further than in 3 s^−1^ and then, again, the curve overlaps with the previous two. For a shear rate of 80 s^−1^, a convex zone extends from the beginning over most of the time of the experiment to allow for the development of an exponential behavior only at the end of the experimental time (orange curves), except in subplot b, in which such exponential development is absent. Notably, the slopes of the exponential parts, once reached, are very similar for each subplot. Such variation is consistent with the starting of the gel effect during the polymerization [92,99]. 

The starting point of each curve in the low-viscosity region in Figure 6 is also located at a higher value of viscosity as the shear rate increases. As the shear rate increases, there is a dampening effect on the viscosity build-up at the beginning of the experiment, which is visible modestly at 3 s^−1^ and extends importantly at 80 s^−1^. The curve for 80 s^−1^ remains lower at higher times as well. On the one hand, there is the influence of the energy from the rheological test itself on the polymerizing system. As the rheometer spindle rotates at a constant velocity, it transmits energy to the system, creating motion and consequently agitation, reminding that no stirrer bar is present as in a conventional large flask experiment. This better mixing for higher shear rates enhances the initial reaction rates so that the growing chains of a certain size start creating entanglement among themselves earlier. This effect translates into an earlier viscosity build-up and finally in molecular diffusion control for the polymerization. On the other hand, higher shear rates are also leaning more toward shear thinning once a polymer is formed, which then lowers the viscosity at least if the shear rate is sufficiently high [100]. Overall, at 80 s^−1^, the better mixing is first winning; then, shear thinning takes over, but in the end, a similar increase in viscosity results. At the lower shear rates, the system first needs to generate sufficient high molar mass chains to obtain a steady growth. Thus, from an “intrinsic” property point of view, the lines at a shear rate of 3 s^−1^ are the most recommended. 

It should be noted that the literature data are less clear in the overall framework of Figure 6, with e.g., a polymerization rate decrease with increasing shear rate [92], a molar mass build-up being hindered with increasing shear rate [101], and a viscosity build-up rate decrease with increasing shear rate [102]. The results in Figure 6 show that these observations are likely only true in certain reaction time regions, benefiting from the application of the developed protocol of Figure 2. 

Furthermore, upon comparing the rows of coformulations in Figure 6, it follows that with only MMA, the viscosity increase is the slowest (subplots a and b). With BA added in the coformulation, a faster increase is obtained (subplot c and d), and with crosslinker (subplot e–h), again, a viscosity acceleration takes place. A closer inspection shows that with the EGDMA crosslinker, the fastest increase results. This is consistent with the MMA conversion data in Figure 4, bearing in mind that the 2% cases are considered. Only with a 5% EGDA crosslinker amount, as explained in Figure 4, one can obtain the opposite effect: thus, the fastest increase with an acrylate-based crosslinker.

From a practical point of view, one can conclude that for the four cases (Cases 3, 5, 7, and 9), the green rheological curves in Figure 6 (3 s^−1^) give more information than the related MMA conversion and thus the GC curves in Figure 4. One still sees the time dependencies in Figure 6, but at the same time, there is a more direct link with the actual macroscopic behavior. Next to that, in practical casting, one has a significant period at high shear rates, which can easily range between 0.01 and 500 s^−1^ based on typical injection speeds and various thicknesses.

### 3.3. Analysis of Mechanical Properties

**Figure 7:**
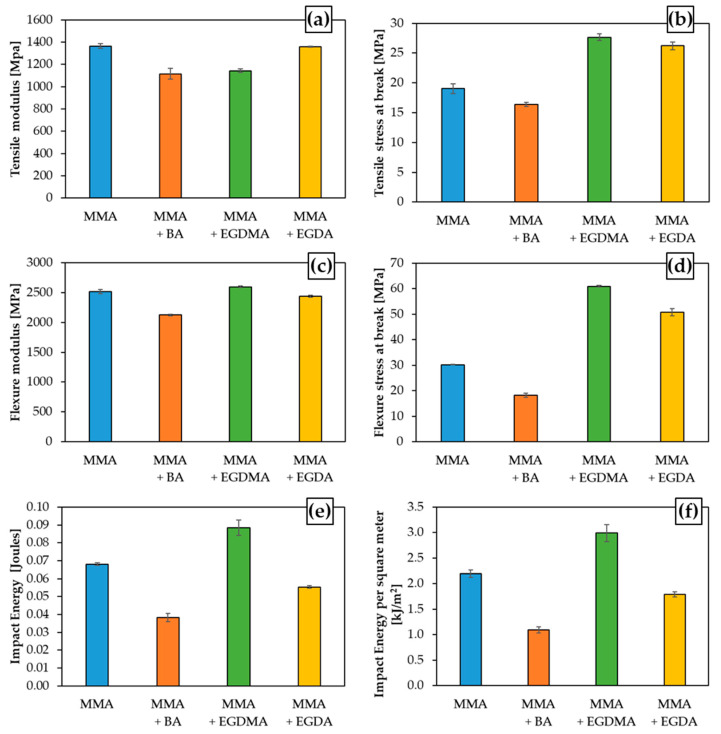
Results of mechanical screening testing, considering the same cases as for the rheological analysis in Figure 6 (see also Table 1): (**a**) Tensile modulus in MPa, (**b**) Tensile stress at break in MPa, (**c**) Flexure modulus in MPa, (**d**) Flexure stress at break in MPa, (**e**) Impact energy in joules; (**f**) Impact energy per square meter at the impact face. The color coding is introduced in Figure 4. Images of broken test samples are provided in the Supporting Information, which also includes the raw data for Figure 7 before statistical averaging.

The mechanical property characterization of the cast materials has been performed considering two types of mechanical testing specimens, namely, dog-bone specimens and unnotched rectangular specimens, as described in Section 2.8. Additional insights have been gained from casting these specimens in silicone molds, as they are not perfectly rigid and they act as a heat sink for the energy generated by the polymerization reaction. This represents a better approximation of the conditions of the actual corrosion casting process, therefore producing results closer to the targeted long-term application. In the present subsection, emphasis is on the tensile test results yielding the tensile modulus and tension at break, the flexure test results delivering the flexure modulus and the tension at break, and the Charpy impact test results allowing measuring the energy dissipated by impact. This impact can be expressed directly or as a quotient including the area of the surface receiving the impact. The joint screening of these three mechanical tests provides a general vision of the stiffness, flexibility, and brittleness of the material, as rarely done for corrosion casting. The raw data and images of the broken test samples are provided in the Supporting Information. Statistical analysis has been performed on these raw data with the final results included in Figure 7.

Figure 7 displays the average mechanical testing values for the cases of Table 1, thus Cases 3, 5, 7, and 9. To remind the reader, Case 3 represents the homopolymerization of MMA, while Case 5 is the copolymerization of MMA and BA, and Case 7 and Case 9 are respectively copolymerizations with EGDMA and EGDA crosslinker. From subplot a, it can be observed that the material with the highest stiffness is the homopolymer of MMA (Case 3, blue bar), being closely followed by the crosslinked material of MMA and EGDA (Case 9, ochre bar), and then the casting materials from Case 7 (MMA/EGDMA) and Case 5 (MMA/BA). The tensile tension at break, as displayed in subplot b, is maximized for the crosslinked materials, thus Case 7 (green bar) and Case 9 (ocher bar). Hence, PMMA proves to be a stiff material if subjected to elongation, and its resistance can be enhanced either by the utilization of EGDMA or EGDA as a crosslinker. As shown in subplot a, for EGDA (Case 9), the highest tensile modulus results through this coformulation design. Crosslinkers allow creating 3D networks of chemical bonds in the polymer structure that are responsible for increasing the resistance but also the rigidity, which can compromise the capacity to withstand stress [103,104,105,106]. Only upon careful selection of the crosslinker, one can overcome this issue as clear from the high yellow bars in both Figure 7a,b.

From Figure 7c,d, flexure mechanical properties can be compared. Subplot c displays the flexure modulus that is similar for the materials from Case 3, Case 7, and Case 9 (blue, green, and ocher bars). In contrast, the material of Case 5 (orange bar), corresponding to the copolymerization of MMA and BA, exhibits a flexure modulus lower than the other three yet not far below. The flexure stress at break reveals a marked difference. The maximal values result for the two crosslinked materials, with the highest one for Case 7 (green bar). Case 3 (blue bar) and Case 5 (orange bar) are considerably inferior regarding the flexure stress at break. It is clear that while the flexure moduli are similar among Case 3 (MMA), Case 7 (MMA/EGDMA), and Case 9 (MMA/EGDA), the increased resistance at break displayed by the materials with either the methacrylate crosslinker or the acrylate crosslinker make Case 7 and Case 9 superior.

The Charpy impact results in Figure 7e,f show that the crosslinker cases are quite different in performance. For Case 7 (MMA/EGDMA), much higher values are obtained. Case 9 still (MMA/EGDA) gives reasonable values, but they are not higher than with MMA only. Hence, if it is desired to maximize all mechanical properties of the four cases in Figure 7, the crosslinking case with EGDMA (Case 7, green bars) is the most interesting. Moreover, upon comparing the rheological data in Figure 6 and the macroscopic property data in Figure 7, it is clear that strong increases of viscosity can be relevant in view of the material design. Specifically, the sharpest increase for Case 7 in Figure 6 (third row) is translated into the best overall performance in Figure 7. However, one should also identify for a given application the minimal macroscopic property value needed, meaning that a lower bar in Figure 7 is not by default a negative result for a final application.

## 4. Conclusions

This experimental study has addressed a formulation matrix comprised of four acrylic monomers, i.e., MMA, BA, EGDMA, and EGDA in view of their long-term corrosion casting potential, considering characterization techniques from the chemical to the material level. The progress of polymerization has been first examined as the MMA conversion variation through GC analysis. The conversion profiles have been compared, and cases of interest have been selected for further rheological and material evaluation, also bearing in mind an analysis of the potential cracking of samples. Four formulations have been selected, one with only MMA, and three copolymerizations being 9:1 MMA/BA, 98:2 MMA/EGDMA, and 98:2 MMA/EGDA.

Rheological tests have been performed for these four cases, all possessing a low initial viscosity and undergoing chemical reaction to deliver highly viscous polymer systems. A novel protocol has been developed for processing the data acquired from constant shear rate tests, which allows visualizing the rheological behavior of a polymerizing system from low to high viscosity. The rheological data allowed to assess the pot-life and the influence of shear rate within the range of corrosion casting shear rates.

Material properties of the four systems have been subsequently determined via tensile, flexure, and Charpy impact tests. An innovative approach has been used to fabricate molds for systems with initial low viscosity and subsequent shrinkage, which allowed the creation of more representative specimens for the mechanical tests. By comparing the results, a relationship between the formulation and the final material properties has been established. The blend MMA/EGDMA (2% crosslinker) exhibited the best performance, in case the desire is to maximize the tensile, flexural, and impact material properties.

Overall, it can be concluded that this study presents an endeavor at combining a formulation range along with rheological and material analysis to establish a more efficient approach for the development of acrylic blends in the scope of manufacturing more sustainable polyacrylic casts. The proposed research strategy is also relevant for future research on polyacrylic materials in general both in industry and academia, mainly contributing to a better linkage of the chemistry and material level.

## Figures and Tables

**Table 1:** (Co)formulation cases of reaction mixtures explored in the present work. Each case is described by the molar percentage composition of the (co)monomer solution, the molar concentration ratio of initiator to monomer (cI_0_/cM_0_), and the mass ratio accelerator to initiator (wA_0_/wI_0_). In all cases, except Case 1, the polymerization was started at room temperature; Case 1 has a starting temperature of 90 °C.

Case	Monomer Molar Percentage				Initiator	Accel. ^1^
	MMA	BA	EGDMA	EGDA	cM_0_/cI_0_	wA_0_/wI_0_
1	100%				100	0
2	100%				100	1
3	100%				100	0.5
4	100%				100	2
5	90%	10%			100	1
6	50%	50%			100	1
7	98%		2%		100	1
8	95%		5%		100	1
9	98%			2%	100	1
10	95%			5%	100	1

^1^ Accel.: accelerator.

**Table 2:** Record of sample vials with crosslinker that cracked (use of “×”) upon quenching in liquid nitrogen, per experiment for gas chromatography and per time of sampling.

Case	2 min	10 min	15 min ^1^	20 min ^2^
7				×
8		×	×	×
9				
10			×	×

^1^ Case 7, as explored first in the present work with respect to Cases 8–10, did not have a sample at 15 min; ^2^ Cases 8–10 were supplemented with an additional sampling at time 15 min due to cracking of the vial of Case 7 at time 20 min.

**Table 3:** Time for the polymerizing mixture to reach a viscosity value of 1000 mPa·s during a rheological test at a constant shear rate ^1^.

Case from Table 1	*T*_0_ = 20 °C No Temperature Control	*T* = 20 °C Constant Temperature
3	≈37 min	≈41 min
5	≈27 min	≈33 min
7	≈17 min	≈19 min
9	≈21 min	≈25 min

^1^ Only shear rates of 1 s^−1^, 3 s^−1^, and 10 s^−1^ were considered here, as the shear rate of 80 s^−1^ was not reaching this limiting value in the maximal time.

## Data Availability

The raw data is available upon reasonable request to the authors.

## Supplementary Materials

The following are available online at https://www.mdpi.com/article/10.3390/ma14226939/s1, Figure S1: Plots illustrating the rheological data processing for Case 3; Figure S2: Plots illustrating the rheological data processing for Case 5; Figure S3: Plots illustrating the rheological data processing for experiment Case 7; Figure S4: Plots illustrating the rheological data processing for experiment Case 9; Figure S5: Conversion, as the percentage BA consumed, versus time for two different formulations (see Table 1 main paper). Case 5 and 6; Figure S6: Conversion, as the percentage EGDMA consumed, versus time for two different formulations (see Table 1 main paper). Case 7 and 8; Figure S7: Conversion, as the percentage EGMA consumed, versus time for two different formulations (see Table 1 main paper). Case 9 and 10; Figure S8: Material tests: (a) specimen after break in a tensile test, (b) several specimens after the tensile test, (c) specimen during a flexure test, (d) several specimens after flexure test; Figure S9: Results of mechanical screening testing, considering the same cases as for the rheological analysis in Figure 6 of the main text (see also Table 1 of the main text).
