# Supplementary material for: Combining Chromatographic, Rheological, and Mechanical Analysis to Study the Manufacturing Potential of Acrylic Blends into Polyacrylic Casts
Source: Materials (Basel). 2021 Nov 17;14(22):6939. doi: 10.3390/ma14226939 (PMC8621424; doi:10.3390/ma14226939)
Supplement: Supplementary file 1 [file materials-14-06939-s001.zip › materials-1461564-supplementary.pdf]

Supporting information for "Combining chromatographic, rheological, and mechanical analysis to study the manufacturing potential of acrylic blends into polyacrylic casts"

Pablo Reyes [1,2,3], Mariya Edeleva [3], Dagmar R. D'hooge [3,4,*], Ludwig Cardon [2] and Pieter Cornillie [1,*]

[1] Laboratory of Morphology, Faculty of Veterinary Sciences, Ghent University, Salisburylaan 133, 9820 Merelbeke, Belgium.; pablo.reyesisaacura@ugent.be (P.R.)

[2] Centre for Polymer and Material Technologies (CPMT), Department of Materials, Textiles and Chemical Engineering, Ghent University, Technologiepark 130, 9052 Zwijnaarde, Belgium.; ludwig.cardon@ugent.be (L.C.)

[3] Laboratory for Chemical Technology (LCT), Department of Materials, Textiles and Chemical Engineering, Ghent University, Technologiepark 125, 9052 Zwijnaarde, Belgium.

[4] Centre for Textiles Science and Engineering (CTSE), Department of Materials, Textiles and Chemical Engineering, Ghent University, Technologiepark 70A, 9052 Zwijnaarde, Belgium.

* Correspondence: dagmar.dhooge@ugent.be (D.R.D.), pieter.cornillie@ugent.be (P.C.)

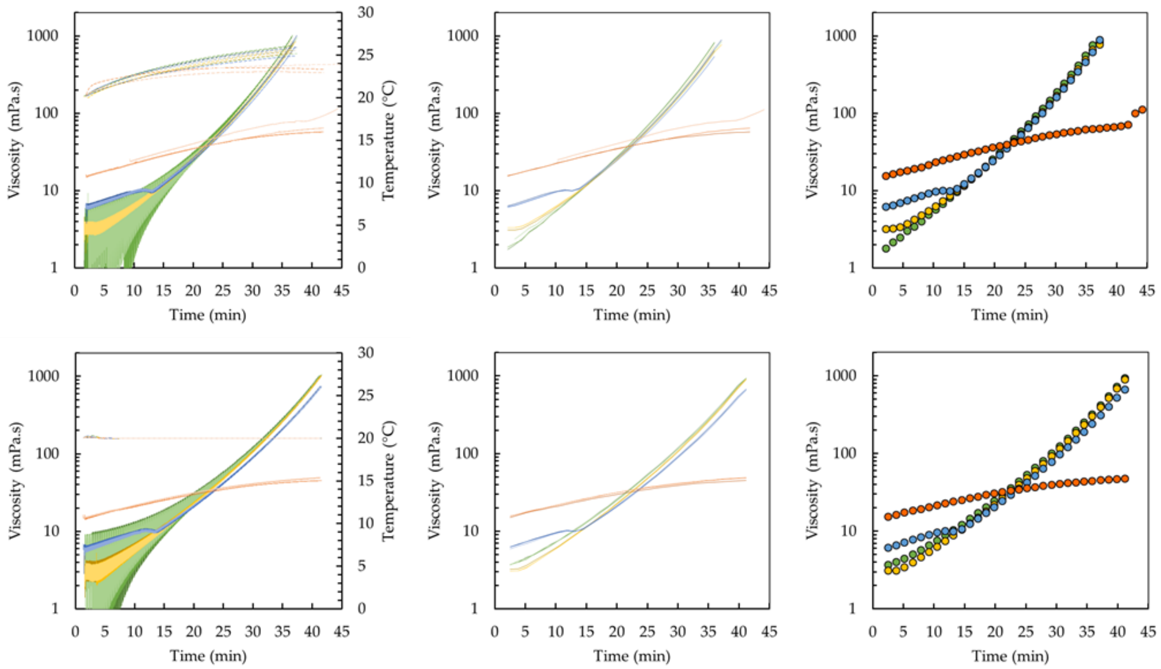

Figure S1. *Plots illustrating the rheological data processing for experiment CASE 3, in which measurements were done by triplicate at each of the following shear rate values: 1 s^{-1} (green), 3 s^{-1} (yellow), 10 s^{-1}(blue) and 80 s^{-1} (orange). The **upper row** corresponds to the experiment starting at 20 °C followed by no temperature control, while the **bottom row** corresponds to the same experiment starting at 20 °C followed by a temperature control to keep the temperature constant . The **left side** plots (viscosity [continuous lines] and temperature [dashed lines] vs time) correspond to the raw data. The **center** plots (viscosity vs time) correspond to the same viscosity values after applying a moving average over a periodic number of points in order to highlight the trend. The **right side** plots (viscosity vs time) correspond to the average value over the measurements for each shear rate value.*

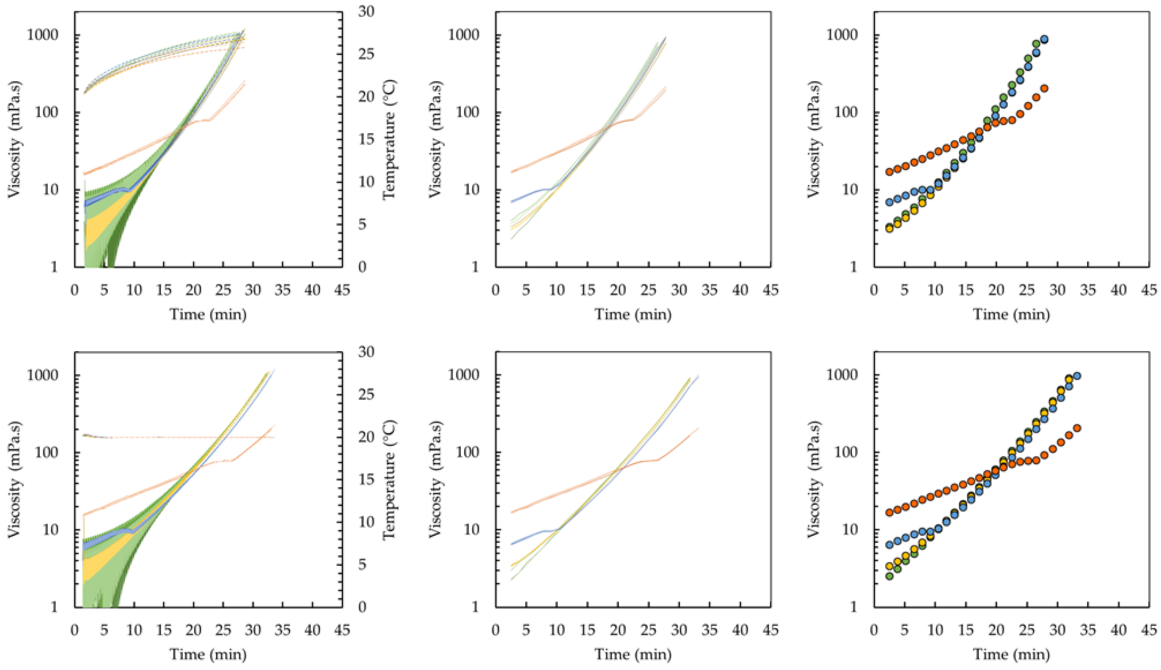

Figure S2. *Plots illustrating the rheological data processing for experiment CASE 5, in which measurements were done by triplicate at each of the following shear rate values: 1 s^{-1} (green), 3 s^{-1} (yellow), 10 s^{-1}(blue) and 80 s^{-1} (orange). The **upper row** corresponds to the experiment starting at 20 °C followed by no temperature control, while the **bottom row** corresponds to the same experiment starting at 20 °C followed by a temperature control to keep the temperature constant . The **left side** plots (viscosity [continuous lines] and temperature [dashed lines] vs time) correspond to the raw data. The **center** plots (viscosity vs time) correspond to the same viscosity values after applying a moving average over a periodic number of points in order to highlight the trend. The **right side** plots (viscosity vs time) correspond to the average value over the measurements for each shear rate value.*

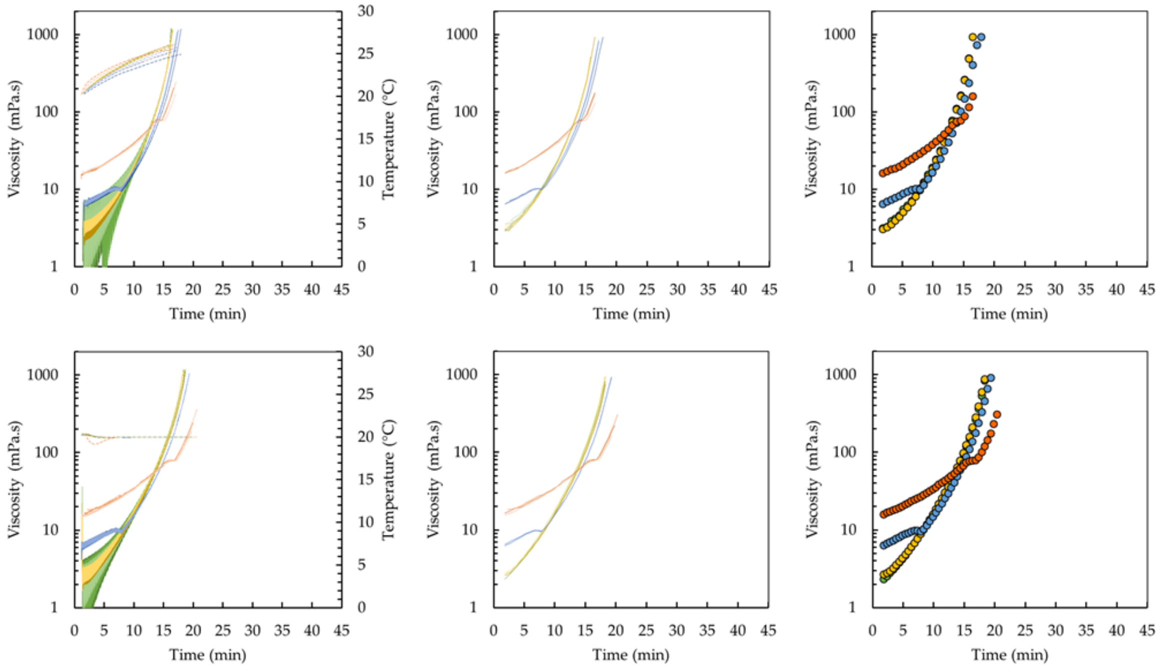

Figure S3. *Plots illustrating the rheological data processing for experiment CASE 7, in which measurements were done by triplicate at each of the following shear rate values: 1 s^{-1} (green), 3 s^{-1} (yellow), 10 s^{-1}(blue) and 80 s^{-1} (orange). The* ***upper row*** *corresponds to the experiment starting at 20 °C followed by no temperature control, while the* ***bottom row*** *corresponds to the same experiment starting at 20 °C followed by a temperature control to keep the temperature constant . The* ***left side*** *plots (viscosity [continuous lines] and temperature [dashed lines] vs time) correspond to the raw data. The* ***center*** *plots (viscosity vs time) correspond to the same viscosity values after applying a moving average over a periodic number of points in order to highlight the trend. The* ***right side*** *plots (viscosity vs time) correspond to the average value over the measurements for each shear rate value.*

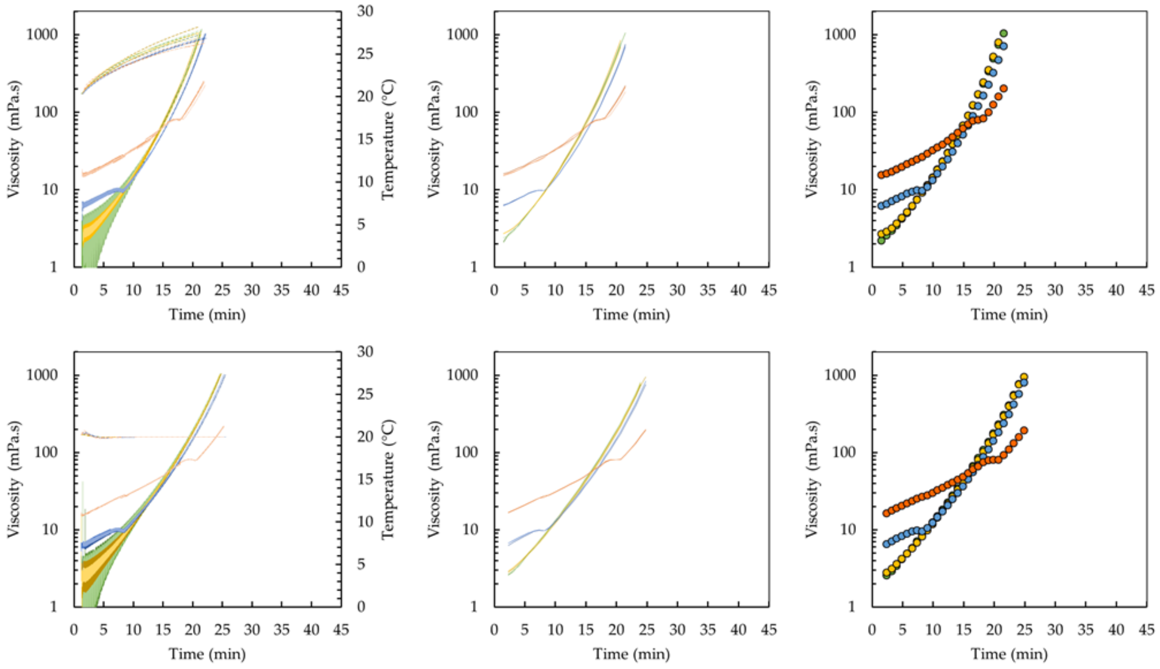

Figure S4. *Plots illustrating the rheological data processing for experiment CASE 9, in which measurements were done by triplicate at each of the following shear rate values: 1 s^{-1} (green), 3 s^{-1} (yellow), 10 s^{-1}(blue) and 80 s^{-1} (orange). The* ***upper row*** *corresponds to the experiment starting at 20 °C followed by no temperature control, while the* ***bottom row*** *corresponds to the same experiment starting at 20 °C followed by a temperature control to keep the temperature constant . The* ***left side*** *plots (viscosity [continuous lines] and temperature [dashed lines] vs time) correspond to the raw data. The* ***center*** *plots (viscosity vs time) correspond to the same viscosity values after applying a moving average over a periodic number of points in order to highlight the trend. The* ***right side*** *plots (viscosity vs time) correspond to the average value over the measurements for each shear rate value.*

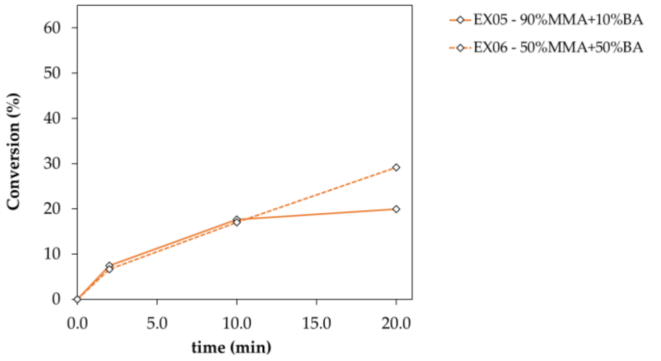

Figure S5. *Conversion, as the percentage BA consumed, versus time for 2 different formulations (see Table 1 main paper). Experiments CASE 5 and CASE 6 starts at room temperature.*

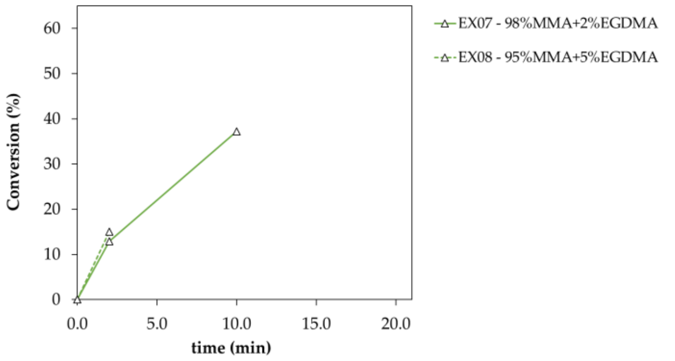

Figure S6. *Conversion, as the percentage EGDMA consumed, versus time for 2 different formulations (see Table 1 main paper). Experiments CASE 7 and CASE 8 starts at room temperature.*

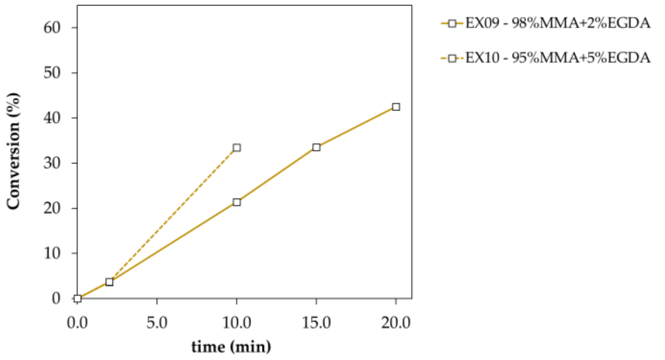

Figure S7. *Conversion, as the percentage EGMA consumed, versus time for 2 different formulations (see Table 1 main paper). Experiments CASE 9 and CASE 10 starts at room temperature.*

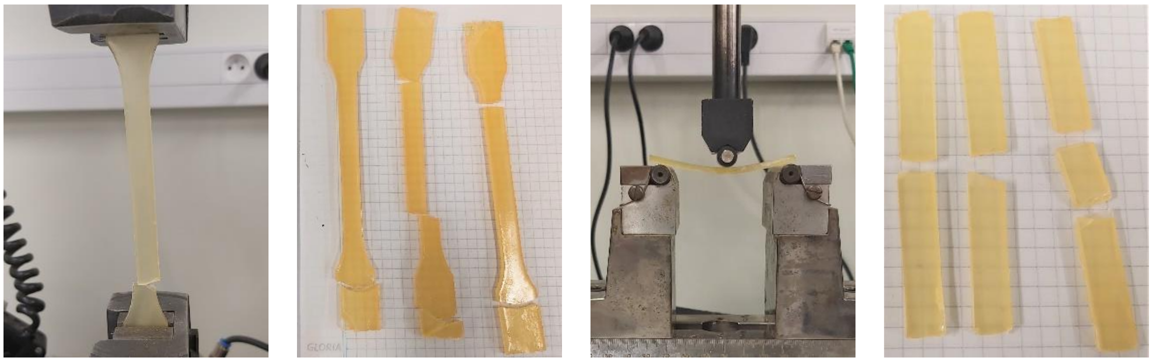

(a) (b) (c) (d)

Figure S8. *Material tests specimens pictures: (a) specimen after break in a tensile test, (b) several specimens after the tensile test, (c) specimen during a flexure test, (d) several specimens after flexure test.*

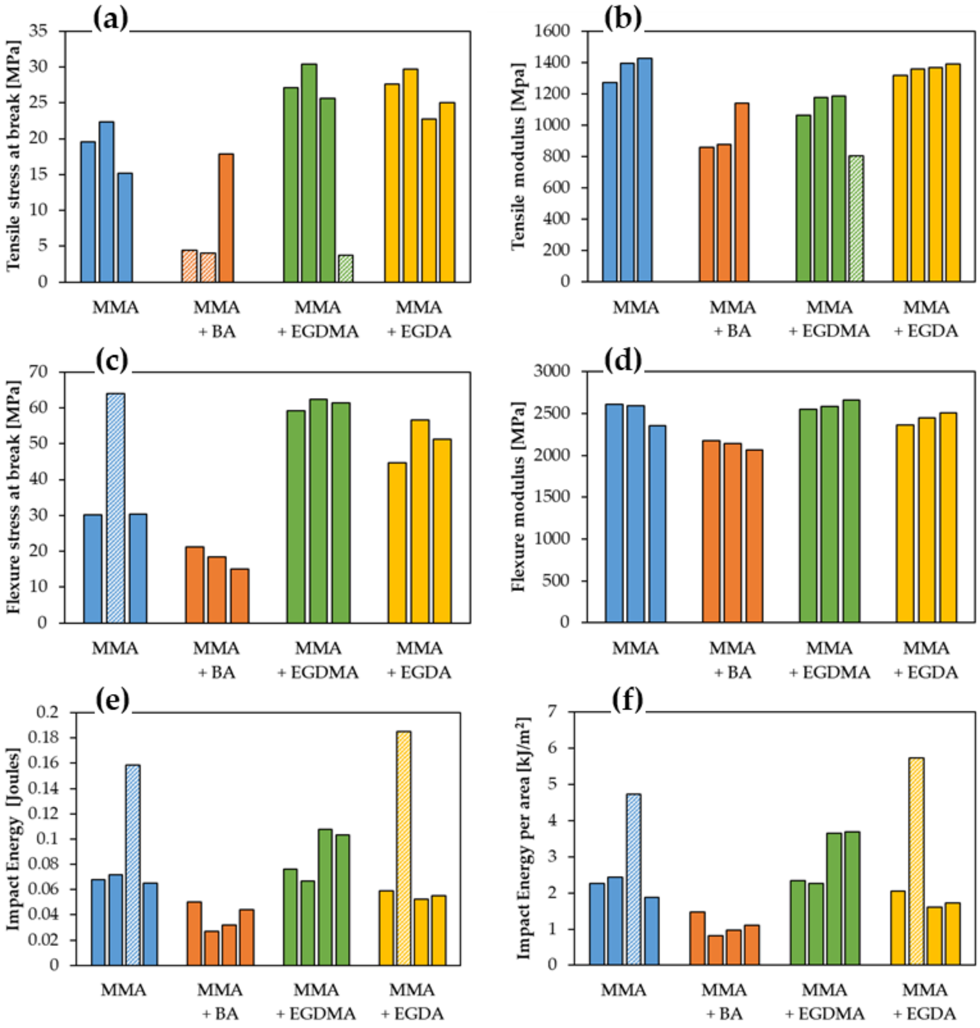

Figure S9. *Results of mechanical screening testing, considering the same cases as for the rheological analysis in Figure 6 of the main text (see also Table 1 of the main text): (a) Tensile modulus in MPa, (b) Tensile stress at break in MPa, (c) Flexure modulus in MPa, (d) Flexure stress at break in MPa, (e) Impact Energy in joules; (f) Impact energy per square meter at the impact face. The color-coding is as introduced in Figure 4 of the main text. Columns filled in dashed pattern indicate results laying out of the general trend, probably due to exceptional casting outcome.*
